# DNA secondary structure is influenced by genetic variation and alters susceptibility to *de novo *translocation

**DOI:** 10.1186/1755-8166-4-18

**Published:** 2011-09-08

**Authors:** Takema Kato, Hidehito Inagaki, Maoqing Tong, Hiroshi Kogo, Tamae Ohye, Kouji Yamada, Makiko Tsutsumi, Beverly S Emanuel, Hiroki Kurahashi

**Affiliations:** 1 Division of Molecular Genetics, Institute for Comprehensive Medical Science, Fujita Health University, Toyoake, Aichi 470-1192, Japan; 2 Division of Human Genetics, The Children's Hospital of Philadelphia, Philadelphia, PA 19104 USA; 3 Department of Pediatrics, University of Pennsylvania School of Medicine, Philadelphia, PA 19104, USA

**Keywords:** Polymorphism, Palindrome, Secondary structure, Hairpin structure, Cruciform structure, Breakpoint, Translocation

## Abstract

****Background**:**

Cumulative evidence suggests that DNA secondary structures impact DNA replication, transcription and genomic rearrangements. One of the best studied examples is the recurrent constitutional t(11;22) in humans that is mediated by potentially cruciform-forming sequences at the breakpoints, palindromic AT-rich repeats (PATRRs). We previously demonstrated that polymorphisms of PATRR sequences affect the frequency of *de novo *t(11;22)s in sperm samples from normal healthy males. These studies were designed to determine whether PATRR polymorphisms affect DNA secondary structure, thus leading to variation in translocation frequency.

****Methods**:**

We studied the potential for DNA cruciform formation for several PATRR11 polymorphic alleles using mobility shift analysis in gel electrophoresis as well as by direct visualization of the DNA by atomic force microscopy. The structural data for various alleles were compared with the frequency of *de novo *t(11;22)s the allele produced.

****Results**:**

The data indicate that the propensity for DNA cruciform structure of each polymorphic allele correlates with the frequency of *de novo *t(11;22)s produced (r = 0.77, *P *= 0.01).

****Conclusions**:**

Although indirect, our results strongly suggest that the PATRR adopts unstable cruciform structures during spermatogenesis that act as translocation hotspots in humans.

## Background

Accumulating evidence indicates that alternative DNA structures (non-B DNA) cause a diversity of genomic rearrangements [1,2]. It is well known that a subset of repeat sequences such as trinucleotide repeats sustain dynamic mutations via DNA secondary structure intermediates leading to their expansion or contraction [3]. The finding that the t(14;18) translocation observed in follicular lymphoma might result from instability of triplex DNA at the breakpoint implies that gross chromosomal rearrangements can also be mediated by non-canonical DNA structures [4,5]. A large-scale survey demonstrates that translocation breakpoints or deletion endpoints in human genetic diseases are consistently found in proximity to predicted non-B DNA structures [6].

Chromosomal translocations have long been thought to be random events. However, recent findings have highlighted two distinct mechanisms that lead to recurrent translocations in humans [7]. A subset of recurrent translocations arises between two homologous regions located on different chromosomes. Robertsonian translocations are mediated by highly repetitive regions on the short arms of the five acrocentric chromosomes, while t(4;8)(p16;p23) translocations result from exchange between two clusters of olfactory-receptor genes on 4p and 8p presumably via homologous recombination [8,9]. Another mechanism is the so-called palindrome-mediated chromosomal translocation [10]. Palindromic AT-rich repeats (PATRRs) were first identified at the breakpoints of the recurrent constitutional t(11;22)(q23;q11) [11-13]. All of the translocation breakpoints are located within the 450 bp PATRR on 11q23 (PATRR11) and the 590 bp PATRR on 22q11 (PATRR22), which do not share sequence homology with one another [14]. The majority of the breakpoints are located at the center of the PATRRs, suggesting that genomic instability of the palindrome center is the etiology of the recurrent translocation [15]. PATRRs also contribute to other recurrent and non-recurrent translocations such as the t(17;22)(q11;q11) [16,17], t(4;22)(q35;q11) [18], t(1;22)(p21.2;q11) [19], and t(8;22)(q24.13;q11.21) [20,21]. Translocation-specific PCR can frequently detect *de novo *t(11;22)s in sperm from normal healthy males [22].

Recently, we also identified *de novo *PATRR-mediated t(8;22)s as well as t(8;11)s by a similar PCR method, suggesting that a considerable proportion of the translocations result from a palindrome-mediated mechanism [21].

Palindromic DNA has the potential to form a secondary structure, an extruded DNA cruciform, through the intra-strand base pairing of adjacent inverted repeat units. A number of palindromic sequences have been identified in the human genome [23], but not all of the palindromes behave as sites for translocation breakpoints. The translocation-associated PATRRs reported so far, share a common structure, 1) a nearly perfect palindrome of several hundred base pairs in length, 2) an AT-rich center and a non-AT-rich region at both ends, 3) another nearby AT-rich region on one side of the PATRR, all of which invoke cruciform structure forming propensity [24]. Indeed, the cloned PATRRs identified at the translocation breakpoints assume a cruciform conformation *in vitro *[25,26]. We propose that the PATRR also adopts a cruciform conformation in living cells, which induces genomic instability leading to translocation formation in humans. In fact, the propensity for secondary structure of the PATRRs on chromosomes 11, 17 and 22 reflects the relative incidence of the relevant chromosomal translocations [27].

In our previous study, we demonstrated that the PATRR11 at the translocation breakpoint often manifests size polymorphisms due to central deletions within the PATRR11, and that this polymorphism affects the frequency of *de novo *t(11;22)s in sperm samples from normal healthy males [28]. Subsequently, we demonstrated that PATRR22 polymorphisms also impact *de novo *translocation frequency [29]. To determine whether PATRR polymorphisms influence secondary structure leading to variation in their translocation frequency, we investigated the secondary structure forming potential of each polymorphic PATRR11 and compared it with its relevant translocation frequency. The results suggest that propensity for secondary structure formation is reflected in the rate of translocations formed.

## Results

### Size and symmetry of the palindromes affect *de novo *translocation frequency

**Figure 1:**
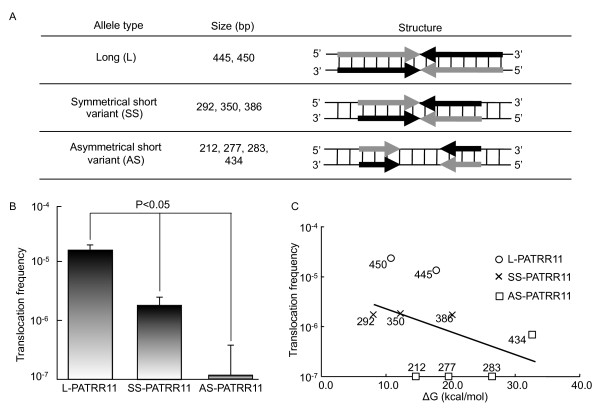
**Polymorphism of the PATRR11 affects *de novo *translocation frequency**. (**A**) Classification of PATRR11s. Arrows indicate each arm of the palindromic sequences. Grey and black arrows indicate complementary strands. (**B**) Size and symmetry of the palindrome affects the *de novo *translocation frequency. Vertical axis indicates *de novo *translocation frequency in sperm. (**C**) Correlation between the translocation frequency and secondary structure-forming propensity of the PATRR analyzed *in silico*. Horizontal axis indicates the free energy necessary for transition from standard double strand DNA to a hairpin structure, while the vertical axis indicates the *de novo *translocation frequency in sperm. No statistically significant correlation between ΔG and translocation frequency was observed (r = 0.372, *P *= 0.323).

To better understand how polymorphic variants of the palindromic sequence affects *de novo *translocation frequency in sperm, we classified the polymorphic PATRR11s into three categories based on the size and symmetry of the palindromic sequences (Figure 1A). The most frequent allele is characterized by a nearly perfect palindromic sequence of 442-450 bps (L-PATRR11). We further grouped minor short variants into symmetric short and asymmetric short PATRR11s (SS-PATRR11 and AS-PATRR11). The size of the SS-PATRR11s and AS-PATRR11s were 212-434 bp.

We attempted to estimate the frequency of *de novo *translocations originating from each PATRR allele. To distinguish the allelic origin of translocation products, we selected individuals heterozygous for PATRR11 polymorphisms for analysis. L-PATRR11 produces *de novo *translocations in approximately 10^-5 ^gametes (1.30-2.11 × 10^-5^). On the other hand, variant PATRR11s generally produce translocations at a lower frequency. For SS-PATRR11, the translocation frequency is about 10-fold lower than that of L-PATRR11 (1.71-1.82 × 10^-6^), while AS-PATRR11s rarely produce *de novo *translocation products (≤6.81 × 10-7) (Table 1, Figure 1B). The differences in translocation frequency were statistically significant between the three groups (*P *= 0.01).

**Table 1:** Potential secondary structure of individual PATRR11 variants by free energy calculation

Type of PATRR11	Nucleotide bp (Accession No.)	G_ds _Kcal/mole	G_stru _Kcal/mole	ΔG Kcal/mole	Translocation frequency
L-PATRR11	445(AF391129)	-392.5	-178.4	17.9	1.32 × 10^-5^
	450(AB235178)	-397.5	-187.9	10.9	2.11 × 10^-5a^

SS-PATRR11	292(AB235183)	-259.8	-121.8	8.1	1.73 × 10^-6^
	350(AB235180)	-314	-144.7	12.3	1.82 × 10^-6a^

AS-PATRR11	386(AB235182)	-335.2	-147.3	20.3	1.71 × 10^-6^
	212(AF391128)	-195.6	-83.1	14.7	<4.05 × 10^-8a^
	277(AB235187)	-247.9	-104.2	19.8	<1.67 × 10^-7^
	483(AB235186)	-252.9	-99.95	26.5	<7.62 × 10^-8^
	434(AB235190)	-380.1	-157.3	32.8	6.81 × 10^-7^

^a ^These values are the mean of 4 (450 bp L-PATRR11 allele), 2 (350 bp SS-PATRR11 allele), and 3 (212 bp AS-PATRR11 allele)samples.

Thus, having determined that the size and symmetry of the PATRR11 appear to determine the frequency of *de novo *t(11;22)s, it seemed reasonable to hypothesize that polymorphisms of the PATRR11 might dictate translocation frequency through their secondary structure-forming propensity.

Therefore, we analyzed the secondary structure-forming propensity of the PATRR by calculating the free energy required for a transition from standard linear double-stranded DNA to intrastrand annealing, or a so-called hairpin structure [19] (Table 1). We then analyzed the correlation between the calculated secondary structure-forming propensity of a given PATRR11 and its *de novo *translocation frequency. The translocation frequency did not correlate with the free energy for hairpin/cruciform formation (r = 0.37, *P *= 0.32) (Figure 1C).

### *In vitro *analysis of cruciform extrusion of PATRR plasmids

**Figure 2:**
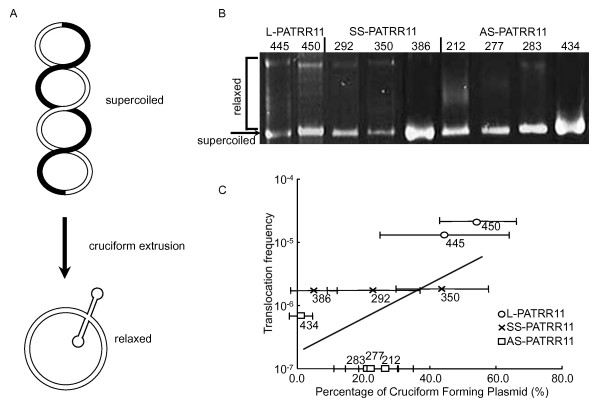
***In vitro *assay for secondary structure formation induced by negative supercoiling**. (**A**) The palindromic region can form cruciform structures in the presence of negative torsion of the double-stranded DNA, which is relieved upon cruciform extrusion. (**B**) EMSA for plasmids during agarose gel electrophoresis. The arrow indicates the position for plasmids with a negative supercoil, while the bracket indicates that for a relaxed plasmid that corresponds to the fraction with cruciform extrusion. In general, relaxed plasmids show up as a ladder consisting of various topoisomers with different linking numbers, which appears as a smear due to the low resolution of gel electrophoresis. (**C**) Correlation between translocation frequency and secondary structure-forming propensity of the PATRR estimated by EMSA. The horizontal axis indicates the percentage of plasmid extruding a cruciform, while the vertical axis indicates the *de novo *translocation frequency in sperm. A linear correlation was observed and was statistically significant (r = 0.71, *P *= 0.03).

We then analyzed the *in vitro *cruciform-forming propensity of the PATRRs using plasmids having various PATRR11s as inserts [25,27]. First we cloned each polymorphic PATRR11 into a plasmid vector and analyzed its propensity for cruciform formation by an electrophoresis mobility shift assay (EMSA). This assay is based on the fact that mobility is retarded when negative superhelical density is relieved by cruciform extrusion (Figure 2A). We extracted plasmid DNA using the triton-lysis method such that cruciform formation during DNA extraction was minimal. To induce cruciform formation, plasmids were incubated for 30 min at 37°C in 100 mM NaCl. The conformation of plasmid DNA was analyzed by band shift on agarose gel electrophoresis.

For L-PATRR11, one distinct band with retarded migration was observed accompanied by a ladder of multiple bands (Figure 2B). We confirmed that the plasmids in the retarded band extruded a cruciform by showing that the band disappeared if the plasmid was digested with T7 endonuclease prior to electrophoresis. This enzyme can cut the four-way junction of cruciform DNA (data not shown). Similar results were obtained in the analysis of SS-PATRR11, whereas AS-PATRR11 did not show such retarded bands. To estimate the percentage of cruciform forming plasmids, we summed the intensity of the retarded bands and calculated their ratio to the sum of all of the bands including the band at the standard negative supercoiled position. The ratio correlated to the frequency of *de novo *translocations for each allele (r = 0.73, *P *= 0.03) (Figure 2C). However, inter-assay variability was significant due to difficulty in the quantification of multiple bands.

**Figure 3:**
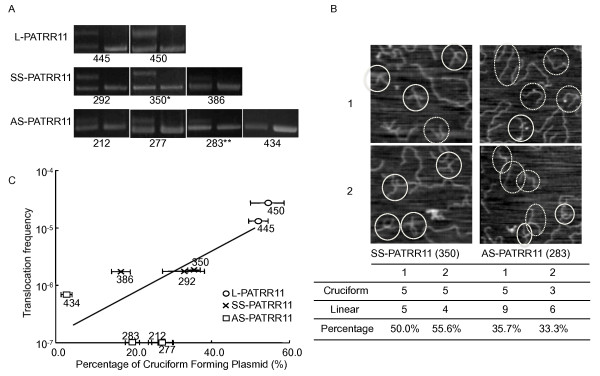
***In vitro *assay for secondary structure formation**. (**A**) EMSA for the plasmid insert upon agarose gel electrophoresis. (right) psoralen crosslinking that can retard the mobility of the cruciform-extruding insert. (**B**) Confirmation of cruciform extruding plasmid inserts by AFM. Images were obtained twice for each sample. Linear or cruciform-extruding inserts were counted. The calculated percentages were approximately equal to the values estimated from EMSA. (**C**) Correlation between translocation frequency and cruciform-forming propensity of the PATRR estimated by EMSA. The horizontal axis indicates the percentage of plasmids extruding a cruciform, while the vertical axis indicates the *de novo *translocation frequency in sperm. Linear correlation was observed which was statistically significant (r = 0.77, *P *= 0.01).

To estimate the prevalence of cruciform extrusion more accurately, EMSA was performed for the plasmid insert only. Since the PATRR cannot maintain a cruciform conformation as short linear DNA, PATRR plasmids were treated with psoralen and ultraviolet light to form covalent crosslinks prior to excision of the plasmid insert by restriction enzyme digestion. We detected a clear retarded band derived from the plasmid insert on standard agarose gel electrophoresis (Figure 3A). We confirmed that the DNAs located in the retarded bands originate from the cruciform by cleavage with T7 endonuclease or by direct observation using atomic force microscopy (AFM) (Figure 3B). The intensity of the retarded band on EMSA correlated well with the translocation frequency (r = 0.77, *P *= 0.01) (Figure 3C).

## Discussion

Our previous study demonstrated that the size and symmetry of polymorphic PATRRs appears to affect the frequency of *de novo *t(11;22)s in sperm samples (Kato et al. 2006, Tong et al. 2010). Here we demonstrate that the size and symmetry of PATRRs reflect their secondary structure propensity. It has been suggested that polymorphic variations affect translocation frequency and induce genomic instability leading to translocation susceptibility. We recently established a model system for generating the t(11;22) using human somatic cell lines [30]. In this system, the endogenous PATRR11 and PATRR22 do not generate t(11;22)s, but two co-transfected plasmids containing a PATRR11 and a PATRR22 generate translocation-like rearrangements only when transfected as cruciform-extruding plasmids. This supports the hypothesis that palindrome-mediated recurrent translocations are facilitated through cruciform extrusion of the two PATRRs.

In our data, the percentage of cruciform DNA for the L-PATRR11 was found to be high, while the translocation frequency was relatively low. One possibility is that we performed these experiments under the conditions that favored for cruciform extrusion to see the difference in cruciform propensity clearly among the PATRR11 variants. It is possible that only a small proportion of PATRRs actually extrude a cruciform in living cells. Another possibility is that the longevity of a cruciform might be transient in living cells. Cruciform extrusion requires strong free negative supercoiling, which could be easily resolved by topoisomerase activity prior to translocation formation.

Among the AS-PATRR11s, only the 434 bp PATRR11 produces translocations, although the cruciform forming propensity of the 434 bp PATRR11 is the lowest. The 434 bp PATRR11 was the longest in length among the AS-PATRR11s we examined in this study. In our previous study, size and symmetry of the PATRR are the important determinants for translocation frequency [28,29]. Size might affect the stability of a cruciform once it forms, or, a cruciform-specific nuclease might more readily recognize and digest a larger cruciform leading to translocation formation.

Although a DNA cruciform is likely to be etiologic for palindrome-mediated translocations, the existence of DNA cruciforms in living cells is still controversial and no direct evidence has yet demonstrated the presence of such a configuration in the context of eukaryotic chromatin [31-34]. Such an energetically unfavorable structure would require sufficient negative superhelicity to stabilize the structure. However, the existence of such a level of negative supercoiling has not yet been proved. Nonetheless, the data in this study indirectly, but strongly imply that PATRRs extrude cruciform structures in living cells. Thus, the question to be answered is when and where such a structure forms and induces a translocation.

We have previously reported sperm-specific occurrence of the t(11;22) translocation in humans [22,35], suggesting that a physiological event during spermatogenesis might be involved in the mechanism of cruciform extrusion and/or structure-dependent instability [36,37]. One way to account for these observations is to postulate that translocations arise during DNA replication. Spermatogenesis engenders a greater number of replications than occur in other somatic tissues or oocytes. The majority of non-recurrent translocations are of paternal origin and *de novo *non-recurrent translocations are often associated with increased paternal age, despite the fact that an age-dependent increase was not observed for the occurrence of the t(11;22) in sperm [38-40]. One possibility is that translocations might occur late in spermatogenesis, when male-specific dynamic changes of chromatin structure take place [41].

In this study, we estimated the ΔG that reflects secondary structures formed by single-stranded DNA. These can be formed within long single-stranded regions of DNA on the lagging-strand template during DNA synthesis. Our data indicate that, similar to the PATRR22, the secondary structure forming propensity of the PATRR11 estimated by its ΔG does not correlate with its translocation frequency [29]. These observations suggest that DNA replication may not significantly contribute to *de novo *translocation formation. This is consistent with the observation that deletions within the PATRR appear to be caused by replication errors, but translocations are not [42,43]. On the other hand, a significant correlate of translocation frequency is observed with the *in vitro *cruciform propensity of PATRR-containing plasmids under torsional constraint. During spermatogenesis standard histones are removed and replaced with protamines at a majority of chromosomal regions. The removal of histones might provide sufficient negative superhelicity to induce cruciform extrusion *in vivo *[44,45]. Such chromatin-remodeling-induced genomic instability deserves further investigation in studies designed to elucidate the mechanism and timing of gross chromosomal rearrangements.

## Conclusions

In this study, a significant association between *de novo *translocation frequency and *in vitro *cruciform forming propensity of the polymorphic alleles of the PATRR11 is observed. Our results indirectly but strongly suggest that the PATRR adopts unstable cruciform structures during spermatogenesis that act as a translocation hotspot in humans.

## Materials and methods

### PCR amplification and cloning of the PATRR

All of the data related to PATRR11 genotype and *de novo *t(11;22) translocation frequency in sperm from normal healthy males have been previously reported [28]. Samples were collected from 2 males who were heterozygous for the L- and SS-PATRR11, while 3 samples were obtained from males heterozygous for L- and AS-PATRR11. Samples from two SS/AS heterozygotes and from one AS/AS heterozygote are also included. All of the donors provided informed consent for further analysis. This study protocol was approved by the Ethical Review Board for Clinical Studies at Fujita Health University.

The PATRR11s were amplified from genomic DNA of the donors by PCR using primers described previously [28]. The plasmids containing the polymorphic PATRR11s were constructed as previously described [26] by TA cloning the PATRR11 PCR products into pT7-blue (Novagen, Madison, WI). The SURE strain (Agilent Technologies, Palo Alto, CA), whose relevant genotype concerning DNA rearrangement and deletion (*recB*, *recJ, sbcC, umuC::Tn5, uvrC*), is known to show increased stability for palindromic sequences, and was used for cloning and propagation of plasmids.

### In silico analysis for secondary structure

Potential secondary structure formed within single-stranded DNA was determined by entering PATRR sequence into the m-fold server http://mfold.rna.albany.edu/?q=mfold/DNA-Folding-Form). A free energy value (G_STRUC_) was obtained. Similarly, free energy values for the same sequence annealed to its complementary strand (G_DS_) were obtained and then halved. Free energy for the formation of secondary structure (ΔG) is calculated as the G_DS _-G_STRUC _difference [19].

### In vitro cruciform extrusion assay

The cruciform-free plasmids were obtained by a denaturation-free, triton-lysis method as previously described [25]. In brief, the *E. coli *cells from a 50 ml culture were dissolved with 10 ml lysis buffer of 50 mM Tris-HCl (pH7.5), 5% sucrose, 1.5 mg/ml lysozyme, 0.1 M EDTA, 25 μg/ml RNase A and 0.75% Triton X-100. The plasmids were extracted without the use of phenol, and purified using an ion-exchange column (QIAGEN, Valencia, CA). The plasmid DNA was precipitated in aliquots with 2-propanol and stored at -30°C until used in an experiment. All of the procedures were performed at 4°C in a cold room to avoid spontaneous cruciform formation during the procedure. To induce cruciform formation, the plasmids were incubated for 30 min at 37°C in 10 mM Tris-HCl (pH 7.5), 0.1 mM EDTA and 100 mM NaCl [27]. The plasmids were cooled on ice before electrophoresis at 50V for approximately 4 hours in a 0.9% agarose gel at 4°C. The gel was stained with ethidium bromide and photographed using the ImageMaster VDS system (GE Healthcare, Diegem, Belgium). Band intensities were quantified using NIH image 1.62 software.

To examine the cruciform conformation in linear DNA, DNA crosslinking was performed by a method similar to that previously described [25]. In brief, plasmid DNA was dissolved in a solution of 4, 5', 8-trimethylpsoralen (100 μg/ml) and exposed to UV light at 365 nm for 5 min. The DNA was digested with the appropriate restriction enzymes to excise the PATRR-containing fragments, purified, and then divided into two aliquots. One half was used for observation by AFM, and the other half was subjected to 2% agarose gel electrophoresis. To confirm that the shifted bands are the result of cruciform extrusion, the plasmid DNA was treated with 5 units of T7 endonuclease I (New England Biolabs, Beverly, MA). Digestion was performed in 20 μl of NEB2 buffer for 40 min. The reaction was performed on ice so as to minimize additional cruciform extrusion during digestion.

### Statistical analyses

Intergroup comparison was performed by one-way analysis of variance, followed by the Mann-Whitney test. Correlations were evaluated with linear straight line regression. In significant difference tests, *P*-values of <0.05 were considered statistically significant.

## Competing interests

The authors declare that they have no competing interests.

## Authors' contributions

TK - Participated in the design of the study, carried out the molecular biology work, and drafted the manuscript. HI - Participated in the design of the study, carried out the molecular biology work. MT - Participated in the design of the study, carried out the molecular biology work. HKO - Participated in the design of the study, carried out the molecular biology work. TO - Participated in the design of the study, carried out the molecular biology work. KY - Participated in the design of the study, carried out the molecular biology work. MT - Participated in the design of the study, carried out the molecular biology work. BSE - Coordinated and conceived the study, being involved in the critical revision of the manuscript for important intellectual content. HKU - Coordinated and conceived the study, participated in the design of the study, drafted the manuscript, being involved in the critical revision of the manuscript for important intellectual content.

All authors have read and approved the final manuscript.
